# Outcomes Following Supracoronary Ascending Aortic Replacement with Aortic Valve Resuspension *versus* Modified Bentall's Operation for Acute Type A Aortic Dissection

**DOI:** 10.21470/1678-9741-2020-0147

**Published:** 2022

**Authors:** Sameer Mohammed, Jayakumar Karunakaran, Vivek V. Pillai

**Affiliations:** 1 Department of Cardiovascular and Thoracic Surgery, Sree Chitra Tirunal Institute for Medical Sciences and Technology, Thiruvananthapuram, Kerala, India.

**Keywords:** Aneurysm, Dissecting, Aortic Valve, Aortic Valve Insufficiency, Dilatation, Bioprosthesis, Heart Valve Prosthesis, Treatment Outcome

## Abstract

**Introduction:**

Type A acute aortic dissection (AAD) remains a challenging cardiac emergency despite the availability of various management strategies. This study compared the outcomes of supracoronary ascending aortic replacement (SCAAR) with aortic valve (AV) resuspension with those of modified Bentall's operation for type A AAD and the progression of aortic regurgitation (AR), long-term dilatation of aortic root and proximal arch, and long-term mortality in SCAAR patients.

**Methods:**

Sixty patients underwent surgery for type A AAD (January 2005 to December 2015). Forty-three patients underwent SCAAR with AV resuspension and 17 underwent modified Bentall's operation. All patients were followed up.

**Results:**

Upon follow-up of SCAAR patients (n=40), there was significant reduction in aortic root size (preoperative 39.3 mm [9.4] *vs*. postoperative 33.1 mm [9.1]; *P*<0.001). Three of these patients worsened to severe AR while others had similar or lesser degree of AR. On comparison between preoperative and postoperative dimensions of all patients (n=53), there was no significant difference in distal ascending aorta size (35.7 mm [8.1] *vs*. 34.4 mm [8.9]; *P*=0.52). However, an increase in descending thoracic aorta size (28.8 mm [7.8] *vs*. 33.7 mm [9.9]; *P*<0.001) was observed. In-hospital and late mortalities for SCAAR *vs*. modified Bentall's procedure were 11.7% (seven patients) (7% [3] *vs*. 23.5% [4]) and 28% (15 patients) (15% [6] *vs*. 69% [9]), respectively.

**Conclusion:**

SCAAR with AV resuspension is a safe surgical option for type A AAD. Preservation of AV is associated with better long-term outcomes and reduced mortality. Modified Bentall's operation may be associated with long-term mortality.

**Table:** 

Abbreviations, acronyms & symbols				
**AA**	**= Ascending aorta**		**CVA**	**= Cerebrovascular accident**
**AAB**	**= Ascending aortic banding**		**CVG**	**= Composite valve graft**
**AAD**	**= Acute aortic dissection**		**DTA**	**= Descending thoracic aorta**
**ACC**	**= Aortic cross-clamp**		**EF**	**= Ejection fraction**
**AR**	**= Aortic regurgitation**		**HR**	**= Hazard ratio**
**AV**	**= Aortic valve**		**LV**	**= Left ventricular**
**CI**	**= Confidence interval**		**PTFE**	**= Polytetrafluoroethylene**
**CPB**	**= Cardiopulmonary bypass**		**SCAAR**	**= Supracoronary ascending aortic replacement**
**CT**	**= Computed tomography**		**SD**	**= Standard deviation**
**CTD**	**= Connective tissue disorders**			

## INTRODUCTION

Acute aortic dissection (AAD) is a surgical emergency necessitating urgent surgery to reduce morbidity and mortality. The reported incidence in literature is three per 100,000 people per year^[1]^. Stanford type A AAD carries a mortality of 50% in 48 hours and 75% in 14 days, if left untreated^[2]^. Surgical management remains the gold standard with operative mortality remaining high (up to 30%) even in experienced centers^[3]^. Repair in type A dissections involves complex strategies ranging from supracoronary ascending aortic replacement (SCAAR) - with or without aortic valve (AV) resuspension for aortic regurgitation (AR) -, modified Bentall's procedure, valve-sparing root operations, frozen elephant trunk technique for thoracic aortic dissection, and total aortic repair^[4]^. Since type A AAD can affect the entire length of the aorta, the native aortic root, the distal part of the ascending aorta (AA) and arch, they may undergo progressive dilatation, sometimes warranting a second procedure later^[5]^. Also, the resuspended AV may develop progressive AR, mandating surgical intervention in the future^[6]^.

Emergency surgery is often performed on a hemodynamically unstable patient to prevent aortic rupture and death^[7]^. Opinions remain divided on the optimal management strategy, with a number of studies validating the aggressive strategy of root replacement with or without AV-sparing procedures and others favoring SCAAR with preservation of AV. Extensive surgical procedures performed on sick patients in an emergency inherently increase the risk of perioperative complications significantly. Also, in developing nations, total corrective repair of aortic dissection poses other challenges like non-availability of composite valve graft (CVG) and paucity of empowered centers with sufficient infrastructure and surgical expertise. There is inconclusive data regarding the progression of AR and long-term changes of aortic root dimensions in patients who underwent AV repair with SCAAR. In this study, we evaluated the preoperative, immediate postoperative, and follow-up data of patients who underwent SCAAR with ascending aortic banding (AAB), neomedia reconstruction, and AV resuspension for Stanford type A AAD. Progression of AR and changes in distal ascending aortic and aortic root dimensions were analyzed. Subgroup analysis of patients with and without connective tissue disorders (CTD) was done. Incidence of aortic dissection is high among patients with CTD and hypertension. Management of these patients is controversial with regard to preservation of aortic root or usage of CVG at the initial setting.

## METHODS

This retrospective study was approved by the institutional ethics committee of our institute (SCT/IEC/899/APRIL-2016). Patients who underwent either modified Bentall's operation or SCAAR and AV resuspension operation for Stanford type A dissection from January 2005 to December 2015 with long-term follow-up were included. A total of 60 consecutive patients were analyzed. Type A dissection was confirmed by presence of intimal flap and entry tear in the AA by two-dimensional echo and computed tomography (CT) scan. Dissection was termed as acute if duration between onset of symptoms and operation was within three weeks. Aortic root and distal AA diameters were calculated pre and postoperatively from CT imaging. Postoperative CT imaging was performed annually after surgery for three years, followed by once in two or three years depending on the findings. For comparative analysis, CT imaging at a three-year follow-up was considered (patients operated till December 2015 were included). Distal AA diameter was calculated just distal to the graft and proximal to the origin of innominate artery.

### Operative Techniques

All procedures were performed within 24 hours of admission. Fifty-one patients were operated within hours of admission following resuscitation, as emergency operation. Chest pain with radiation to the back was the most common presenting symptom. All operations were performed by two surgeons who have expertise in aortic surgeries. During our initial years of study, we preferred modified Bentall's operation for many patients. However, in the more recent years, we have increasingly performed valve-preserving SCAAR. Furthermore, patients with dissection up to leaflet commissures, dissection appearing in previously existing aneurysm, and unfavorable leaflet morphology (bicuspid valve, calcified valve, deformed valve, injured valve) have undergone modified Bentall's operation. Intraoperatively, the choice of either replacement or repair technique was also based on surgeon's preference and likelihood of successful repair. All patients were cooled down to 18 °C. All procedures were performed on aortic cross-clamp (ACC) and limited period of circulatory arrest of 1-2 min to inspect arch for any entry tears. Any reentry tears were managed with hemiarch procedure. Those who underwent hemiarch procedure were excluded from the study.

In modified Bentall's operation, AV and dissected AA were replaced with CVG designed by suturing a mechanical new generation, low-profile tilting disc valve (TTK Chitra Heart Valve, TTK Healthcare, Chennai, India) in 14 patients; mechanical bileaflet valve (St. Jude Medical, Masters Series, Puerto Rico, United States of America) in two patients; bioprosthetic pericardial leaflet prosthesis - Bio Bentall (PERIMOUNT Magna - Edwards Lifesciences, Irvine, United States of America) in one patient to a bovine collagen-coated polyester graft (InterGard, InterVascular, France or Gelweave, Vascutek Terumo, Scotland, United Kingdom).

The procedure of SCAAR, AAB, neomedia reconstruction with AV resuspension was performed as described below. Cardiopulmonary bypass (CPB) was instituted by femoral artery (predominantly in about 70% of patients when there is no dissection involving it) or axillary artery (either directly or through a 7-mm graft) and right atrial cannulation. After ACC was placed, AA was opened, true lumen was identified, and cardioplegic arrest was achieved by ostial cardioplegia (St. Thomas or Custodial HTK cardioplegia). Dissected AA was resected. Proximal false lumen was obliterated by neointimal reconstruction with a piece of polytetrafluoroethylene (PTFE) strip, sized and shaped to conform with the dissected area in the aortic root. Human thrombin fibrin sealant (EVICEL Fibrin Sealant, Ethicon, United States of America or TISSEEL, Baxter pharmaceuticals, United States of America) was applied to hold the layers together and reinforced by approximating the layers together using 5-0 polypropylene suture.

AV resuspension was performed using pledgetted 4-0 polypropylene with pledget placed outside aorta and sutured in a horizontal mattress fashion. AA was replaced with an appropriately sized collagen-coated polyester graft. Anastomosis was performed by incorporating the entire thickness of aortic wall, including the neomedia, using 4-0 polypropylene, which was further reinforced with PTFE strip placed outside aorta. Human thrombin sealant was applied at all anastomotic sites to prevent postoperative bleeding.

AAB was performed with a broad strip of approximately 2 cm in width positioned around distal AA, just distal to the graft at the site of ACC. This can be performed by placing a 2-cm long tube graft railroaded over the main graft as a "sleeve" prior to performing distal anastomosis. Once distal anastomosis is finished, the sleeve is moved over the anastomosis to the distal aorta. The other technique uses a 2-cm wide PTFE strip to wrap around the distal AA. In both techniques, the band is anchored to the underlying aorta to prevent distal migration and occlusion of arch vessels.

### Statistical Methods

Univariate analysis was done, and the results were presented as percentages for categorical variables and as mean and standard deviation for continuous variables. For bivariate analysis, Fisher's exact test was used for binary variables. The Student's *t*-test/Welch's *t*-test was used to compare the groups for continuous variables after *F*-test. Paired *t*-test was used to compare preoperative and postoperative measurements. Survival was estimated using the Kaplan-Meier curve. Cox proportional hazards regression analysis was performed to evaluate the effect of available perioperative data on survival rates. All statistical data were analyzed using IBM Corp. Released 2012, IBM SPSS Statistics for Windows, Version 21.0, Armonk, NY: IBM Corp. software and *P*-values <0.05 were considered significant.

### Power Calculation

There were seven deaths out of 43 patients in the SCAAR group and nine deaths out of 17 patients in the Bentall group at the end of one year. When the mortality ratio in the SCAAR group is 16.3% and in the Bentall group is 52.9%, considering an alpha error of 5%, the power based on normal approximation is 84.16%. The power was calculated with the help of OpenEpi 3.01 software (Open Source Epidemiologic Statistics for Public Health).

## RESULTS

### Patients' Demographic Characteristics

A total of 60 patients were analyzed. They were divided into two groups based on the surgical procedure. General characteristics, operative and intraoperative variables, and postoperative characteristics are depicted in Table 1. The left ventricular (LV) function of patients with AR was categorized as mild (ejection fraction [EF] 46-54%), moderate (EF 36-45%), and severe (EF<36%) LV dysfunction. AR was moderate to severe in 76.6% of the patients (n=46). There was a significant difference in terms of age at presentation, hypertension, CPB time, ACC time, and mean follow-up (years) between the two groups. Mean CPB time was 141.4±35.9 minutes. The mean ACC time was 97.3±20.6 minutes. Nine patients (15%) had adverse neurological event in the form of delayed awakening (not awake > 72 h), stroke, paraplegia, and coma. Mean creatinine value (mg/dL) was higher in the postoperative period (1.67 [0.67] *vs*. 1.37 [0.89]). Serum creatinine levels remained elevated postoperatively in patients with higher levels in the preoperative period. There were no new neurological events in patients with old history of cerebrovascular accident (CVA) following surgery.

**Table 1:** General characteristics, operative and intraoperative variables, and postoperative characteristics of the study population.

Variable	SCAAR (n=43, 71.7%)	Modified Bentall (n=17, 28.3)	Total (N=60)	*P*-value*
Age (years), mean (SD)	48.2 (11.6)	40.2 (11.3)	45.9 (11.9)	0.019
Male, n (%)	28 (65.1)	11 (64.7)	39 (65)	1.000
Connective tissue disorder, n (%)	9 (20.9)	8 (47.1)	17 (28.3)	0.059
Hypertension, n (%)	28 (65.1)	6 (35.3)	34 (56.7)	**0.046**
Old CVA, n (%)	2 (4.7)	1 (5.9)	3 (5)	1.000
Preoperative creatinine (mg/dl), mean (SD)	1.40 (0.98)	1.31 (0.64)	1.37 (0.89)	0.730
LV function, n (%)				
Good	38 (88.4)	12 (70.6)	50 (83.3)	
Mild dysfunction	3 (7)	4 (23.5)	7 (11.7)	
Moderate dysfunction	1 (2.3)	0 (0)	1 (1.7)	
Severe dysfunction	1 (2.3)	1 (5.9)	2 (3.3)	
Mild to severe LV dysfunction, n (%)	5 (11.6)	5 (29.4)	10 (16.7)	0.128
AR grade, n (%)				
No	2 (4.7)	1 (5.9)	3 (5)	
Trivial	1 (2.3)	0 (0)	1 (1.7)	
Mild	9 (20.9)	1 (5.9)	10 (16.7)	
Moderate	17 (39.5)	6 (35.3)	23 (38.3)	
Severe	14 (32.6)	9 (52.9)	23 (38.3)	
Moderate to severe AR grade, n (%)	31 (72.1)	15 (88.2)	46 (76.7)	0.310
Emergency surgery, n (%)	39 (90.7)	12 (70.6)	51 (85)	0.101
Cardiopulmonary bypass time (min), mean (SD)	133.09 (31.36)	162.47 (38.88)	141.42 (35.89)	**0.003**
Aortic cross-clamp time (min), mean (SD)	90.16 (17.22)	115.53 (17.29)	97.35 (20.62)	**< 0.001**
Postoperative creatinine (mg/dl), mean (SD)	1.60 (0.53)	1.85 (0.98)	1.67 (0.67)	0.238
Adverse neurological event, n (%)	7 (16.3)	2 (11.8)	9 (15)	1.000
In-hospital mortality, n (%)	3 (7)	4 (23.5)	7 (11.7)	0.092
Follow-up (years), mean (SD)	6.42 (3.4)	3.50 (2.58)	4.52 (3.8)	0.09
Preoperative aortic root (mm), mean (SD)	39.26 (9.45)	42.59 (8.43)	40.20 (9.23)	0.210
Postoperative aortic root (mm), mean (SD)	33.13 (9.1)	29.46 (5.19)	32.23 (8.42)	0.175
Preoperative DTA at hiatus (mm), mean (SD)	29.72 (7.5)	26.59 (8.21)	28.83 (7.77)	0.161
Postoperative DTA at hiatus (mm), mean (SD)	34.97 (10.45)	29.92 (7.01)	33.71 (9.89)	0.112
Preoperative distal ascending aorta (mm), mean (SD)	35.98 (8.45)	35.06 (7.45)	35.72 (8.13)	0.697
Postoperative distal ascending aorta (mm), mean (SD)	35.08 (9.69)	32.46 (5.44)	34.43 (8.86)	0.361

AR=aortic regurgitation; CVA=cerebrovascular accident; DTA=descending thoracic aorta; LV=left ventricular; SCAAR=supracoronary ascending aorta replacement; SD=standard deviation

* Fisher's exact test and t-test.

Three patients had malperfusion. One of them underwent fenestration near renal artery for renal malperfusion and the other two underwent aortoiliac stenting for lower limb malperfusion immediately following primary surgery. Nine patients had additional procedures, eight had coronary artery bypass grafting (most commonly, single graft to right coronary artery), and one had mitral valve replacement (severe mitral regurgitation for mitral valve prolapse).

There were seven deaths in early postoperative period. In-hospital mortality was 11.7%, with the SCAAR group [7%, n=3] having lower rate compared to the modified Bentall group [23.5%, n=4]. Death causes include cardiogenic shock (n=4), coma (n=1), bleeding (n=1), and multi-organ dysfunction syndrome (n=1). The mean age of the patients who died is 46±12 years. All patients who had in-hospital mortality were operated as emergency. Multivariate analysis could not be performed as the number of deaths were small.

The mean follow-up period was 4.5±3.8 years. Preoperative and postoperative dimensions of aortic root, distal AA (distal to graft), and descending thoracic aorta (DTA) at hiatus level were compared in all patients who underwent surgery and also in SCAAR and modified Bentall groups separately (Table 2). There was significant reduction in aortic root dimensions following SCAAR surgery with no further increase on follow-up.

**Table 2:** Preoperative and postoperative comparison of aortic diameters at various levels in patients who underwent surgery for aortic dissection.

Variable	Preoperative	Postoperative	*P*-value*
Aortic root, mean (SD)			
SCAAR operation	39.3 (9.4)	33.1 (9.1)	**< 0.001**
Modified Bentall's procedure	42.6 (8.4)	29.5 (5.2)	**< 0.001**
All patients	40.2 (9.2)	32.2 (8.4)	**< 0.001**
Distal ascending aorta, mean (SD)			
SCAAR operation	36 (8.4)	35.1 (9.7)	
Modified Bentall's procedure	35.1 (7.4)	32.5 (5.4)	
All patients	35.7 (8.1)	34.4 (8.9)	0.521
DTA at hiatus, mean (SD)			
SCAAR operation	29.7 (7.5)	35 (8.4)	
Modified Bentall's procedure	26.6 (8.2)	30 (7.1)	
All patients	28.8 (7.8)	33.7 (9.9)	**< 0.001**

DTA=descending thoracic aorta; SCAAR=supracoronary ascending aorta replacement; SD=standard deviation

* Paired t-test.

Progression of AR was followed up in all patients who underwent AV resuspension along with SCAAR. Out of the seven patients who had moderate AR in the immediate postoperative period, one patient progressed to severe AR. Two of the patients (11%) who had only mild AR in the early postoperative period progressed to severe AR. The remaining patients in the mild AR group continued to have similar or lesser degree of AR. Conjecturally, those with higher degree of AR in the early postoperative period tend to worsen, thus mandating late reintervention.

### Survival Analysis

During follow-up, there were 15 (28%) deaths among 53 patients. Late mortality during follow-up also depicted poor survival among modified Bentall patients with mortality rate of 69% (n=9 of 13 survivors), whereas patients who have undergone SCAAR have only 15% (n=6 of 40 patients). Causes of late deaths include neurological causes (n=3, stroke=1, intracranial bleed=2), perioperative death while undergoing reintervention (n=2), chest pain (n=3), sudden cardiac death (unexplained) (n=5), and morbid illness (n=2). Multivariate analysis was done to predict risk factors of long-term mortality (Table 3). None of the factors were shown to predict long-term mortality. Though long-term mortality is higher in the modified Bentall group as compared to the SCAAR group, it failed to show association on Cox proportional hazard model. Larger numbers are required to predict risk factors of mortality.

**Table 3:** Predictors of long-term mortality.

Variable	Long-term outcome HR (95% CI)*
Female sex	1.24 (0.41 - 3.78)
Connective tissue disorder	0.45 (0.1 - 2.09)
Hypertension	1.05 (0.35 - 3.14)
Emergency surgery	1.34 (0.37 - 4.81)
Modified Bentall's surgery	3.11 (0.84 - 11.54)
Mild to severe LV function	2.15 (0.65 - 7.13)
Age (years)	1.01 (0.96 - 1.06)
Preoperative creatinine > 1.37	1.28 (0.41 - 3.97)
CBP time > 141.42	1.49 (0.41 - 5.39)
ACC time > 97.35	1.47 (0.36 - 6.04)

ACC=aortic cross-clamp; CI=confidence interval; CPB=cardiopulmonary bypass; HR=hazard ratio; LV=left ventricular

* Cox proportional hazard model.

**Fig. 1:**
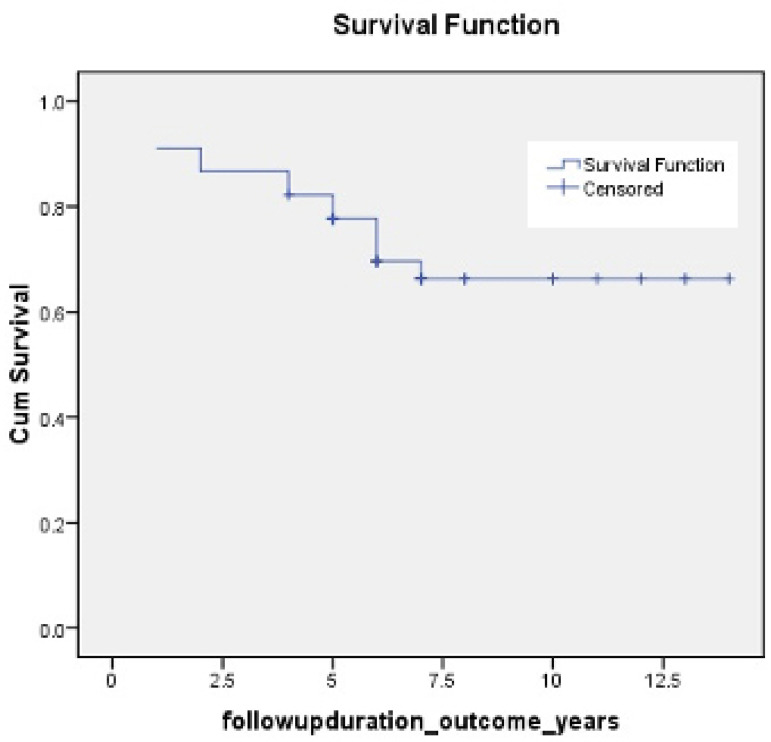
Overall survival of all patients who underwent type A aortic dissection repair.

Almost 53% of deaths (including in-hospital mortality) in the modified Bentall group have occurred during the first year following surgery, probably related to the management of anticoagulation and postoperative valve replacement related complications (arrhythmias or heart blocks). Overall survival of all patients at end of one, five, and ten years is 91%, 78%, and 66%, respectively, with mean average survival of 11 years (Figure 1).

**Fig. 2:**
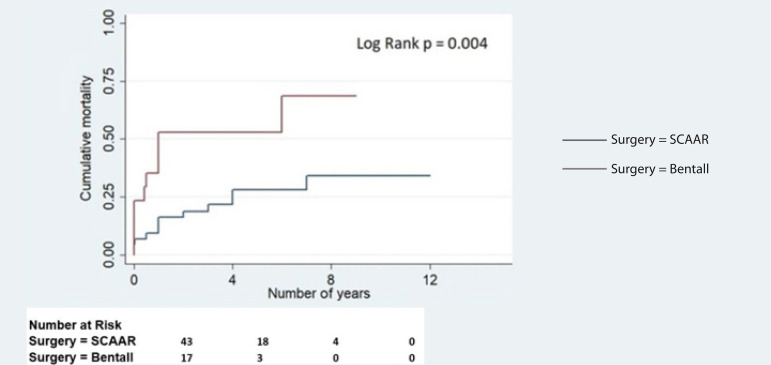
Kaplan-Meier curves showing actuarial survival difference between supracoronary ascending aorta replacement (SCAAR) with aortic valve resuspension patients vs. the modified Bentall group.

Six patients underwent reintervention during the follow-up period. The reintervention procedures include debranching and thoracic endovascular aortic repair (n=2), aortic root replacement (n=2), DTA aneurysm repair (n=1), and fenestration (n=1). Two patients succumbed to death perioperatively during these procedures. The rest were kept on medical follow-up to monitor for AR progression and management. Kaplan-Meier analysis for long-term survival was calculated between the two surgical groups (Figure 2).

Comparison analysis among patients with and without CTD revealed significant differences in age, hypertensive status, and preoperative and postoperative serum creatinine levels. Patients with CTD presented with higher degrees of AR as compared to the rest (88.2% *vs*. 72%, respectively) (Table 4). Though there was minimal dilatation of aortic root preoperatively in the CTD group, there was no difference postoperatively.

**Table 4:** Comparison of various variables between the CTD and non-CTD groups.

Variable	CTD group (n=17, 28.3%)	Non-CTD group (n=43, 71.7%)	Total (N=60)	*P*-value*
Age (years), mean (SD)	35.7 (7.3)	50 (11)	45.9 (11.9)	< 0.05
Male, n (%)	12 (70.5)	27 (62.7)	39 (65)	0.56
Hypertension, n (%)	4 (23.5)	30 (69.8)	34 (56.7)	0.001
Preoperative creatinine (mg/dl), mean (SD)	1.01 (0.3)	1.51 (0.9)	1.37 (0.89)	0.005
Preoperative creatinine > 1.4 mg/dl, n (%)	1 (5.8)	18 (41.8)	19 (31.6)	0.006
Mild to severe LV dysfunction, n (%)	4 (23.5)	6 (13.9)	10 (16.7)	-
Old CVA, n (%)	0 (0)	3 (6.9)	3 (5)	0.64
Emergency operation, n (%)	14 (82.4)	37 (86)	51 (85)	1.0
SCAAR with AV resuspension, n (%)	9 (52.9)	34 (79.1)	43 (71.6)	0.08
Modified Bentall's operation, n (%)	8 (47.1)	9 (20.9)	17 (28.3)	0.08
Cardiopulmonary bypass time (min), mean (SD)	133 (21.5)	145 (39.9)	141.42 (35.89)	0.15
Aortic cross-clamp time (min), mean (SD)	96.7 (17.7)	97.6 (21.8)	97.35 (20.62)	0.86
Postoperative elevated creatinine (> 1.5 mg/dl), n (%)	3 (17.6)	21 (48.8)	24 (40)	0.02
Preoperative aortic root (mm), mean (SD)	41.1 (11.7)	39.9 (8.2)	40.20 (9.23)	0.654
Postoperative aortic root (mm), mean (SD)	32.1 (8.7)	32.3 (8.4)	32.23 (8.42)	0.949

AV=aortic valve; CTD=connective tissue disorder; CVA=cerebrovascular accident; LV=left ventricular; SCAAR=supracoronary ascending aorta replacement; SD=standard deviation

* Fisher's exact test and t-test.

## DISCUSSION

Surgery for type A aortic dissection is challenging despite advances in surgical techniques, cerebral protection strategies, and perioperative management. Its complexity is attributed to the fact that aorta is the main vessel arising from left ventricle, to its proximity to arch vessels, to the fragility of the dissected tissues, the necessity of periods of total circulatory arrest, and the requirement of a bloodless operative field. If the surgery is delayed, it can lead to aortic rupture, severe AR, and malperfusion of various end-organs of the body^[8]^. Furthermore, it can compromise coronary and cerebral circulation. Hence, early identification and prompt management play a pivotal role.

Even though literature is replete with studies on extent of repair, choice of approach, and perioperative morbidity and mortality, the ideal surgical approach still remains controversial^[9]^. Studies by Colli et al. and Russo et al. concluded that perioperative risk factors play an important role on patient outcomes^[10,11]^. Merkle et al. concluded that the incidence of perioperative complications increased significantly with extent of surgical approach^[12]^. Many surgeons have adopted the strategy of limiting the complexity of surgery with the primary goal being improvement of early operative outcome rather than long-term postoperative consequences^[7,13]^. This can be achieved by limiting the extent of distal repair with or without conservative root repair techniques^[9,14]^.

Preservation of native AV with AV resuspension can be safely performed when root is not excessively dilated and extent of dissection is above sinotubular junction^[14]^. Limiting extent of distal repair includes either hemiarch replacement or replacement of AA alone. Cabasa and Pochettino have suggested remaining conservative at the level of root, but to perform more extensive repair at level of arch^[9]^. Our approach has been relatively conservative, and the extent of distal repair is individualized to each patient.

Studies by Rice et al. and Crawford et al. suggested that isolated AA replacement with distal ACC was limited only to limited DeBakey II dissections, because they found the risk of distal anastomotic disruption and malperfusion of arch vessels high with increased incidence of strokes^[15]^. We have employed placement of ACC for most of the patients and none of our patients had distal anastomotic site dilatation or disruption. This favorable outcome is probably due to placement of AAB with PTFE strip around the distal anastomotic site which supports it till it heals. Comparison of preoperative and postoperative dimensions of distal AA distal to the graft remained similar (35.7 vs. 34.4 mm) without any significant dilatation.

Adverse neurological events can occur preoperatively (due to involvement of arch vessels) or perioperatively. Though the adverse neurological events are slightly high (15%) in our study, results are comparable with literature of 11-29% in various series^[16]^. Few of these patients have cerebrovascular risk factors like diabetes mellitus and cigarette smoking and few others have presented in late stages with hypotension. Though antegrade cerebral perfusion is preferred to retrograde perfusion through femoral arteries, our higher event rate could also be attributed to these confounders apart from surgical technique.

Traditionally, dissections are operated on deep hypothermic circulatory arrest with or without antegrade cerebral perfusion. ACC is avoided by many surgeons due to fear of damaging clamped aorta and causing micro debris embolization, which can compromise cerebral protection. Despite clamp application, our neurological events were not drastically high^[17]^. This has been achieved due to maintenance of cerebral and lower body perfusion, examination of arch with limited circulatory arrest to ensure no reentry tears promoting progression of dissection, keeping clamp time as minimum as possible, deep hypothermia (18 °C), and careful manipulation of aorta. Perfusion of the rest of the body while performing arch repair can have favorable outcomes in terms of end-organ protection. Song et al. have shown lower hepatic and renal enzymes in patients who underwent aortic arch repair under moderate hypothermia with intermittent lower body perfusion providing more effective end-organ protection^[18]^. Cerebral malperfusion remains the most important cause of cerebral injury intraoperatively^[19]^. Studies by Kim et al. and Merkle et al. have shown that total arch replacement can cause more CVA^[12,20]^.

The most commonly utilized site for arterial cannulation is the femoral artery because of its rapid accessibility, and it is the preferred site in many centers. Potential dangers include retrograde debris embolization, extension of dissection of intimal flap, expansion of the false lumen, and possible organ malperfusion^[9]^.

Total arch repair is associated with higher morbidity and mortality compared to less invasive approaches as reflected in studies by Kim et al. and Colli et al.^[10]^. Opponents of less aggressive surgery emphasize on persistence of residual false lumen, which poses as a significant risk factor for future potential reintervention. Resection of all reentry tears have to be confirmed for permitting complete thrombosis of false lumen^[21,22]^. Song et al. have shown significant higher incidence of partial thrombosis of residual false lumen in DTA with less aggressive strategies compared with total arch replacement with poor long-term survival and need for reopening for bleeding. Corroborative evidence was seen in this study that DTA measurements at hiatus were significantly higher postoperatively at follow-up compared to preoperative measurements (33.7 *vs*. 28.8 mm, respectively), which indicates expansion of residual false lumen. There were no patients with bleeding episodes which required redo surgery^[23]^. Lenos et al. found that performance of aortic repair in type A AAD by experienced aortic surgeons have better outcomes and higher curative rates^[24]^. Two surgeons who have experience in aortic surgery performed all repairs at our center.

In developing nations where accessibility and resources are limited and with few aortic centers, provision for best care to all patients is challenging. Hemostats have also improved results by significantly reducing postoperative bleeding and the need for blood transfusions. Procedures on ACC enable surgeons to perform the procedure accurately without fear of cerebral and lower body malperfusion.

Subgroup analysis of patients with and without CTD revealed that CTD patients were younger at presentation, whereas the non-CTD group had higher incidence of hypertension and higher serum creatinine level in preoperative and postoperative periods. A recent study has shown significantly higher rate of postoperative complications in patients with preoperatively elevated serum creatinine^[25]^.

Patients with CTD, presented with higher degrees of regurgitation probably related to underlying condition involving aortic root. Follow-up data of SCAAR patients showed good outcomes with majority of patients having no progression of AR. These findings are corroborating with conclusions of Tang et al. where AV resuspension improves valve competency even in patients with moderate to severe AR at presentation with good outcomes and the overall survival remains unchanged. Though studies are available on progression of AR, progression particularly in those with CTD needs to be studied with a larger sample size to draw conclusions.

Consensus exists regarding rates of mortality, with most centers reporting low in-hospital mortality. In-hospital mortality in this study was 11.7%, which is comparable to the figures in the International Registry of Acute Aortic Dissections. Higher incidence of in-hospital or 30-day mortality was seen with aggressive surgical approaches and longer ACC times in literature. Corroboratively, ACC time has been significantly associated with higher incidence of postoperative complications, bleeding, and transfusion requirements. Survival analysis has shown significant mortality in the Bentall group with lower long-term survival reflecting aggressive surgical strategy affecting long-term outcomes.

### Limitations

Larger sample size is required to predict various factors affecting in-hospital and late mortalities. Specific studies must be undertaken in patients with CTD and aortic dissection in large numbers. Studies of similar nature with valve-sparing root replacements have to be encouraged.

## CONCLUSION

Although the choice and extent of surgical approach remain largely debated, SCAAR with AAB and AV resuspension is a safe option with good surgical outcomes. Preservation of native AV should be attempted whenever possible even in cases with severe AR as it has better long-term outcomes. Modified Bentall's procedure may be associated with higher long-term mortality as compared to SCAAR, though procedure per se might not be a significant risk factor for mortality.

**Table:** 

Authors' roles & responsibilities	
SM	Substantial contributions to the conception and design of the work; and the acquisition, analysis, and interpretation of data for the work; drafting the work; final approval of the version to be published
JK	Substantial contributions to the design of the work; and the interpretation of data for the work; revising the work critically for important intellectual content; final approval of the version to be published
WP	Substantial contributions to the conception and design of the work; and the interpretation of data for the work; drafting the work; final approval of the version to be published

## References

[1] Grau JB, Kuschner CE, Ferrari G, Wilson SR, Brizzio ME, Zapolanski A (2015). Effects of a protocol-based management of type A aortic dissections. J Surg Res.

[2] Fukui T (2018). Management of acute aortic dissection and thoracic aortic rupture. J Intensive Care.

[r3] Afifi RO, Sandhu HK, Leake SS, Rice RD, Azizzadeh A, Charlton-Ouw KM (2016). Determinants of operative mortality in patients with ruptured acute type A aortic dissection. Ann Thorac Surg.

[4] Matalanis G, Ip S (2019). A new paradigm in the management of acute type A aortic dissection: total aortic repair. J Thorac Cardiovasc Surg.

[5] Li D, Ye L, He Y, Cao X, Liu J, Zhong W (2016). False lumen status in patients with acute aortic dissection: a systematic review and meta-analysis. J Am Heart Assoc.

[6] Roselli EE, Loor G, He J, Rafael AE, Rajeswaran J, Houghtaling PL (2015). Distal aortic interventions after repair of ascending dissection: the argument for a more aggressive approach. J Thorac Cardiovasc Surg.

[7] Nishida H, Tabata M, Fukui T, Takanashi S (2016). Surgical strategy and outcome for aortic root in patients undergoing repair of acute type A aortic dissection. Ann Thorac Surg.

[8] Erbel R, Aboyans V, Boileau C, Bossone E, Bartolomeo RD, Eggebrecht H (2014). 2014 ESC guidelines on the diagnosis and treatment of aortic diseases: document covering acute and chronic aortic diseases of the thoracic and abdominal aorta of the adult. The task force for the diagnosis and treatment of aortic diseases of the European society of cardiology (ESC). Eur Heart J.

[9] Cabasa A, Pochettino A (2016). Surgical management and outcomes of type A dissection-the Mayo clinic experience. Ann Cardiothorac Surg.

[10] Colli A, Carrozzini M, Francescato A, Galuppo M, Comisso M, Toto F (2018). Acute DeBakey type I aortic dissection without intimal tear in the arch: is total arch replacement the right choice?. Interact Cardiovasc Thorac Surg.

[11] Russo CF, Mariscalco G, Colli A, Santè P, Nicolini F, Miceli A (2016). Italian multicentre study on type A acute aortic dissection: a 33-year follow-up†. Eur J Cardiothorac Surg.

[12] Merkle J, Sabashnikov A, Deppe AC, Zeriouh M, Maier J, Weber C (2018). Impact of ascending aortic, hemiarch and arch repair on early and long-term outcomes in patients with Stanford A acute aortic dissection. Ther Adv Cardiovasc Dis.

[13] Ro SK, Kim JB, Hwang SK, Jung SH, Choo SJ, Chung CH (2013). Aortic root conservative repair of acute type A aortic dissection involving the aortic root: fate of the aortic root and aortic valve function. J Thorac Cardiovasc Surg.

[14] Tang PC, Badami A, Akhter SA, Osaki S, Lozonschi L, Kohmoto T (2017). Efficacy of aortic valve resuspension in establishing valve competence in acute type A dissections. Ann Thorac Surg.

[15] Rice RD, Sandhu HK, Leake SS, Afifi RO, Azizzadeh A, Charlton-Ouw KM (2015). Is total arch replacement associated with worse outcomes during repair of acute type A aortic dissection?. Ann Thorac Surg.

[16] Czerny M, Schoenhoff F, Etz C, Englberger L, Khaladj N, Zierer A (2015). The impact of pre-operative malperfusion on outcome in acute type A aortic dissection: results from the GERAADA registry. J Am Coll Cardiol.

[17] Dumfarth J, Kofler M, Stastny L, Plaikner M, Krapf C, Semsroth S (2018). Stroke after emergent surgery for acute type A aortic dissection: predictors, outcome and neurological recovery. Eur J Cardiothorac Surg.

[18] Song SW, Yoo KJ, Shin YR, Lim SH, Cho BK (2013). Effects of intermittent lower body perfusion on end-organ function during repair of acute DeBakey type I aortic dissection under moderate hypothermic circulatory arrest. Eur J Cardiothorac Surg.

[19] Furukawa T, Uchida N, Takahashi S, Yamane Y, Mochizuki S, Yamada K (2017). Management of cerebral malperfusion in surgical repair of acute type A aortic dissection. Eur J Cardiothorac Surg.

[20] Kim JB, Chung CH, Moon DH, Ha GJ, Lee TY, Jung SH (2011). Total arch repair versus hemiarch repair in the management of acute DeBakey type I aortic dissection. Eur J Cardiothorac Surg.

[21] Hirotani T, Nakamichi T, Munakata M, Takeuchi S (2003). Routine extended graft replacement for an acute type A aortic dissection and the patency of the residual false channel. Ann Thorac Surg.

[22] Urbanski PP, Siebel A, Zacher M, Hacker RW (2003). Is extended aortic replacement in acute type A dissection justifiable?. Ann Thorac Surg.

[23] Song SW, Chang BC, Cho BK, Yi G, Youn YN, Lee S (2010). Effects of partial thrombosis on distal aorta after repair of acute DeBakey type I aortic dissection. J Thorac Cardiovasc Surg.

[24] Lenos A, Bougioukakis P, Irimie V, Zacher M, Diegeler A, Urbanski PP (2015). Impact of surgical experience on outcome in surgery of acute type A aortic dissection. Eur J Cardiothorac Surg.

[25] Eghbalzadeh K, Sabashnikov A, Weber C, Zeriouh M, Djordjevic I, Merkle J (2018). Impact of preoperative elevated serum creatinine on long-term outcome of patients undergoing aortic repair with Stanford A dissection: a retrospective matched pair analysis. Ther Adv Cardiovasc Dis.

